# Hybrid endoscopic submucosal dissection: An alternative resection modality for large laterally spreading tumors in the cecum?

**DOI:** 10.1186/s12876-021-01766-w

**Published:** 2021-05-05

**Authors:** Xiang-Yao Wang, Ning-Li Chai, Ya-Qi Zhai, Long-Song Li, Zan-Tao Wang, Jia-Le Zou, Yong-Sheng Shi, En-Qiang Linghu

**Affiliations:** Department of Gastroenterology and Hepatology, First Medical center of Chinese PLA General Hospital, No. 28 Fuxing Road, Beijing, 100853 China

**Keywords:** Laterally spreading tumors, Endoscopic submucosal dissection, Hybrid endoscopic submucosal dissection, Cecum

## Abstract

**Background:**

Endoscopic resection for large, laterally spreading tumors (LSTs) in the cecum is challenging. Here we report on the clinical outcomes of hybrid endoscopic submucosal dissection (ESD) in large cecal LSTs.

**Methods:**

We retrospectively reviewed data from patients with cecal LSTs ≥ 2 cm who underwent ESD or hybrid ESD procedures between January of 2008 and June of 2019. We compared the baseline characteristics and clinical outcomes, including procedure time, the en bloc and complete resection rates, and adverse events.

**Results:**

A total of 62 patients were enrolled in the study. There were 27 patients in the ESD group and 35 patients in the hybrid ESD group, respectively. Hybrid ESD was more used for lesions with submucosal fibrosis. No other significant differences were found in patient characteristics between the two groups. The hybrid ESD group had a significantly shorter procedure time compared with the ESD group (27.60 ± 17.21 vs. 52.63 ± 44.202 min, *P* = 0.001). The en bloc resection rate (77.1% vs. 81.5%, *P* = 0.677) and complete resection rate (71.4% vs. 81.5%, *P* = 0.359) of hybrid ESD were relatively lower than that of the ESD group in despite of no significant difference was found. The perforation and post-procedure bleeding rate (2.9% vs. 3.7%, *P* = 0.684) were similar between the two groups. One patient perforated during the ESD procedure, which was surgically treated. One patient in the hybrid ESD group experienced post-procedure bleeding, which was successfully treated with endoscopic hemostasis. Post-procedural fever and abdominal pain occurred in six patients in the ESD group and five patients in the hybrid ESD group. One patient in the ESD group experienced recurrence, which was endoscopically resected.

**Conclusion:**

The results of this study indicate that hybrid ESD may be an alternative resection strategy for large cecal LSTs with submucosal fibrosis.

## Background

Endoscopic treatment is becoming more widely used for colorectal laterally spreading tumors (LSTs). Surgery can typically be avoided for lesions without deep submucosal invasion if the endoscopic resection is successful [1–3]. However, certain tumor locations make endoscopic resection more challenging, such as cecal LSTs. These represent a small proportion of overall colonic LSTs [4, 5].

Endoscopic mucosal resection (EMR) is a traditional and widely used therapeutic modality for treating LSTs [6, 7]. However, en bloc resection for lesions ≥ 2 cm is difficult to achieve with EMR. The resection is usually conducted in a piecemeal fashion, which makes the histological evaluation imprecise and has a higher local recurrence rate, as demonstrated in previous studies [8, 9]. En bloc resection should be undertaken for lesions with a higher risk of submucosal invasion, like granular-type LSTs with a large nodule or depression and nongranular-type LSTs [2, 10]. Endoscopic submucosal dissection (ESD), which can achieve higher en bloc and complete resection rates, is time-consuming and has a higher adverse event rate. More feasible and effective endoscopic resection strategies are needed to treat large cecal LSTs.

Hybrid ESD is one of the many modified techniques developed to overcome these problems. Hybrid ESD, which combines the advantages of EMR and ESD, uses a snare to remove the remaining lesion after submucosal dissection [11–13]. A meta-analysis reported that hybrid ESD was less effective than ESD for colorectal neoplasia [12, 14–16]. However, some of the research on hybrid ESD contained scared and refractory cases, which may underestimate this technique [17, 18]. Recent research, including a randomized controlled trial, demonstrated that hybrid ESD had similar efficacy and safety in the treatment of colorectal neoplasia as ESD. Hybrid ESD can also be used as a rescue method for difficult cases [19–21]. While this technique may be suitable for cecal LSTs ≥ 2 cm, there is limited data on hybrid ESD in large cecal LSTs to guide the clinical approach.

The purpose of this study is to evaluate the feasibility, efficacy, and safety of hybrid ESD for the treatment of large cecal LSTs.

## Methods

### Patients and study design

Data on patients with LSTs treated by endoscopic resection in a single, academic, tertiary center were retrospectively collected from endoscopic and clinical databases between January of 2008 and June of 2019. The information collected included baseline characteristics and procedure outcomes. A pre-procedural evaluation was performed for all lesions, including chromoendoscopy with indigo carmine and narrowband image (NBI) endoscopy, to classify the gross type and pit pattern. The gross type was divided into two groups: granular (LST-G) and non-granular (LST-NG), according to Kudo’s classification [7]. The LST-G includes homogeneous (LST-G-H) and nodular mixed (LST-G-NM) types. The LST-NG includes flat-elevated (LST-NG-FE) and pseudodepressed (LST-NG-PD) types. The exclusion criteria included a lesion size of < 2 cm or a lesion resected by EMR, a preprocedural evaluation suggesting a deep, invasive lesion [2, 22, 23], a lesion with a difficult-to-treat location, and non-lifting signs that suggest deep, submucosal invasive cancer.

**Fig. 1:**
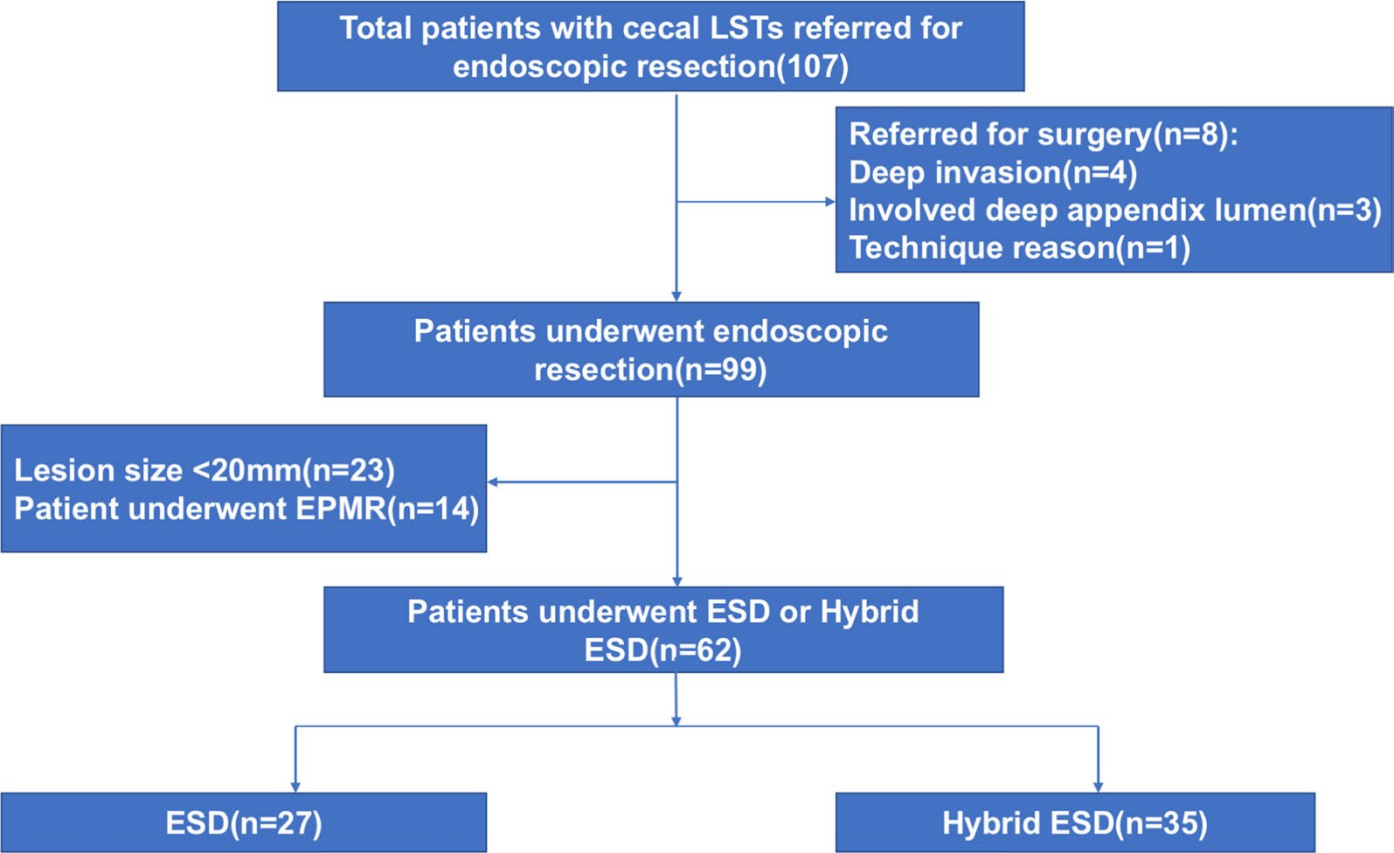
Study flow chart

We consulted a specialist for the patients undergoing antithrombotic therapy to determine when to withdraw or replace medications. A flow chart of this study is shown in Fig. 1. All subjects were treated on an inpatient basis, after they were informed of the benefits and risks of endoscopic intervention. Written informed consent was obtained before endoscopic procedures were performed. Professor EQ.LH and NL.C led the team to conducted the study and performed the procedures.  This study was approved by the Ethics Committee of the Institutional Review Board of Chinese PLA General Hospital, complying with the Declaration of Helsinki.

### Endoscopic resection

Endoscopic resection, including ESD and hybrid ESD, was performed using a single-channel endoscope (PCF-Q260J, Olympus, Tokyo, Japan) with a transparent plastic cap on its tip. A VIO200D electrosurgical unit was used (ERBE, Tubingen, Germany). The cutting current was the ENDO CUT mode (effect 3, cut duration 2, and cut interval 2) and the dissection current was the FORCED COAG mode (effect 4 and max Watts 50). 

### The ESD technique

A solution of epinephrine and normal saline (1:10,000) containing methylene blue was injected below the lesion to provide a submucosal cushion. After the submucosal injection, circumferential incisions and submucosal dissections were performed using a dual or IT knife (Olympus, Tokyo, Japan), and the lesion was completely removed from the muscle layer without any snaring. Visible vessels in the defect after tumor resection were treated with hemostatic forceps. Clips (Boston Scientific, Marlborough, USA) were used to close the defect after resection.

### The Hybrid ESD technique

**Fig. 2:**
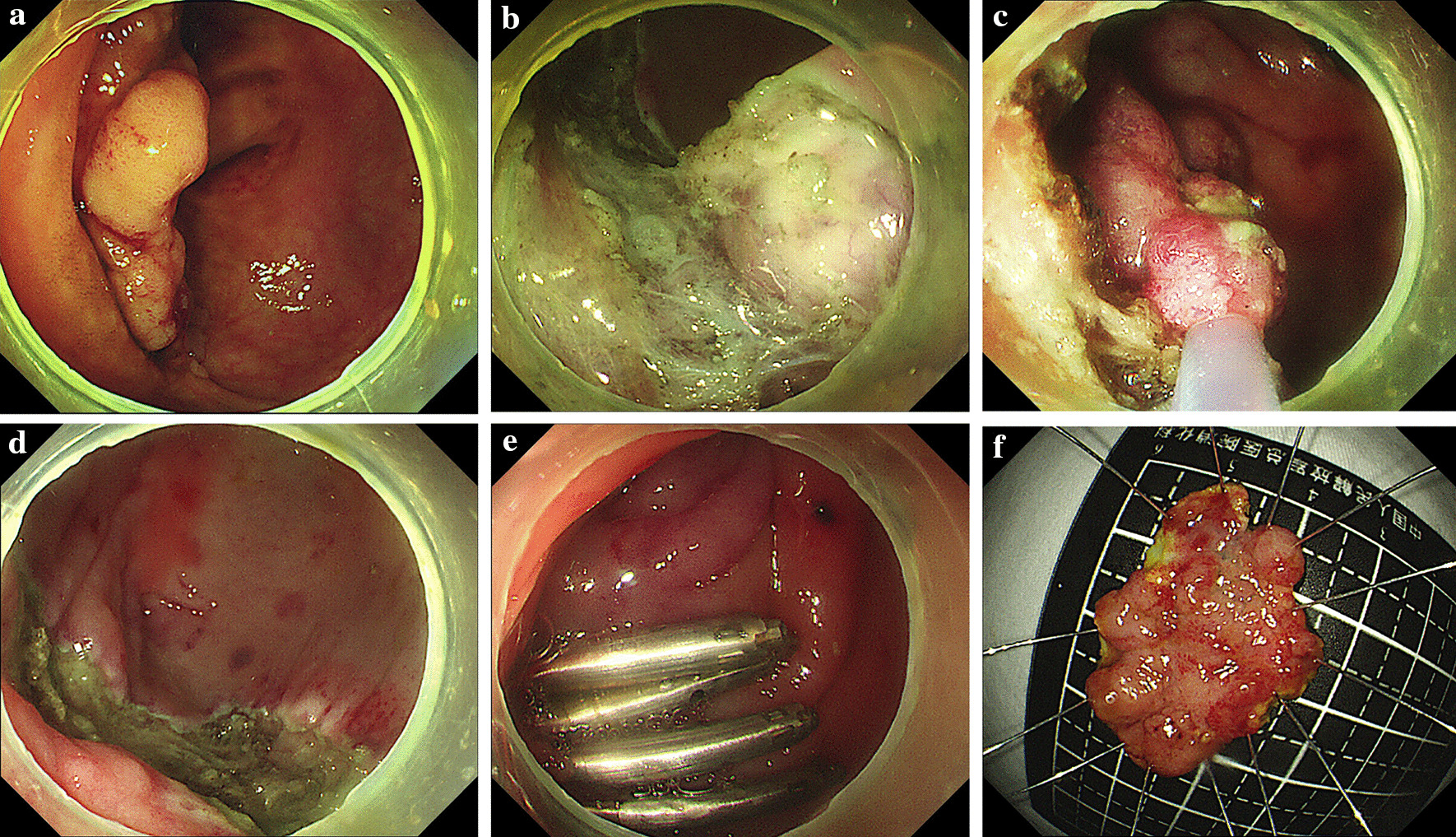
A hybrid ESD for a laterally spreading tumor resection. **a** A granular-type, laterally-spreading tumor involving the inferior lip of the ileocecal valve. **b** Submucosal dissection after a circumferential incision in the lesion. **c** A snaring resection was used to remove the lesion. **d** A defect after the resection. **e** Clips were used to close the defect. **f** The specimen was resected en bloc

The submucosal injection and circumferential incision of the hybrid ESD were performed as described above. Snaring was performed after the submucosal dissection using a 3 × 6 cm polypectomy snare (Cook, Winston-Salem, USA) to remove the remainder of the lesion. The other procedure steps was similar to the ESD methodology described above. The procedure is detailed in Fig. 2.

Procedure time was measured from the submucosal injection to completion of the lesion resection. Endoscopic en bloc resection was defined as the resection of the entire tumor in one piece, as determined by endoscopic observation. Complete resection (R0) was defined as a resection with tumor-free vertical and lateral margins, as determined by histopathological evaluation.

The degree of submucosal fibrosis was evaluated at the time of the injection of solution of epinephrine and normal saline (1:10,000) containing methylene blue during ESD; The classification of submucosal fibrosis was as followed: F0, no fibrosis, which manifests as a transparent layer; F1, mild fibrosis, which appeared as a white web-like structure in the submucosal layer; and F2, severe fibrosis, which appeared as a white muscular structure without a transparent layer in the submucosal layer [24].

### Histopathological evaluation

The resected specimens were fixed on a plate to measure lesion size. They were then fixed in a formalin solution, sliced at 2 mm intervals, and stained with hematoxylin and eosin (H&E) and immunohistochemical (IHC). The histopathological assessments, including histological type, lateral and vertical resection margins, and invasion depth, were based on the World Health Organization’s gastrointestinal epithelial neoplasia classifications [25].

### Complications

Post-procedural bleeding was defined as clinically-evident hematochezia within 15 days after the endoscopic resection that required hemostatic medications, a blood transfusion, surgery, angiography, or endoscopic hemostasis to control [26]. Perforation was diagnosed during the procedure or afterward if free air was visible on an abdominal plain radiograph. Patients reporting abdominal pain and fever after the procedure required medical examination and treatment.

### Subgroup analysis

We performed subgroup analyses by lesion morphology to compare the ESD and hybrid ESD characteristics and outcomes for different types of cecal LSTs.

### Statistical analysis

Continuous variables, presented as a mean with a standard deviation (SD) or a median with a range, were analyzed with a student’s t-test or Mann–Whitney U test. Categorical data were expressed as numbers and percentages, and a Chi-square test was used for analysis. A multivariable logistic regression analysis was used to analyze the factors associated with a shorter procedure time. A *P*-value of < 0.05 was considered statistically significant. All data analyses were conducted with IBM SPSS statistical software, version 23.0 (IBM Corp., Armonk, NY, USA).

## Results

### Baseline characteristics of patients and lesions

Table 1 shows the baseline characteristics of the patients and lesions in both groups. A total of 62 patients were enrolled in the final analysis. There were 27 patients in the ESD group and 35 patients in the hybrid ESD group. LST-G-NM was the most common type of lesion in both groups. Appendiceal orifice or ileocecal valve involvement was noted in 11 (40.7%) lesions in the ESD group and seven (20.0%) lesions in the hybrid ESD group. Histopathological results showed that one (3.7%) patient in the ESD group and two patients (5.7%) in the hybrid ESD group had submucosal invasion cancer. Two patients in each group underwent antithrombotic therapy (three patients took aspirin and one patient took clopidogrel). All patients stopped taking the medicine seven days before the procedure. There were no significant differences in sex, age, BMI, lesion size, endoscopic type, involvement range, and histopathology between the two groups. The hybrid ESD group had a higher submucosal fibrosis rate than the ESD group (*P* = 0.033).

**Table 1:** Baseline characteristics

	ESD (n = 27)	Hybrid ESD (n = 35)	*P-*value
Patients			
Sex (male/female)	8/19	13/22	0.535
Age, median (range), years	63 (34–75)	61 (39–82)	0.955
BMI, median (range)	23.7 (19.1–31.1)	24.4 (16.9–33.2)	0.422
Lesions			
Size, median (range), mm	25 (20–62)	23 (20–42)	0.104
Endoscopic type, n (%)			0.464
LST-G-H	10 (37.0%)	9 (25.7%)	
LST-G-NM	14 (51.9%)	18 (51.4%)	
LST-NG-FE	3 (11.1%)	6 (17.2%)	
LST-NG-PD	0 (0.0%)	2 (5.7%)	
Appendiceal orifice or Ileocecal valve involvement, n (%)			0.142
Appendiceal orifice	5 (18.5%)	4 (11.4%)	
Ileocecal valve	5 (18.5%)	1 (2.9%)	
Both Ileocecal valve and appendiceal orifice	1 (3.7%)	2 (5.7%)	
Histopathology, n (%)			0.517
Adenoma with low grade dysplasia	17 (63.0%)	27 (77.1%)	
Adenoma with high grade dysplasia	7 (25.9%)	5 (14.3%)	
Intramucosal cancer	2 (7.4%)	1 (2.9%)	
Submucosal invasion cancer	1 (3.7%)	2 (5.7%)	
Antithrombotic medications^b^, n (%)			0.788
Antiplatelet agents	2 (7.4%)	2 (5.7%)	
Anticoagulants	0 (0.0%)	0 (0.0%)	
Submucosal fibrosis, n (%)			0.033^a^
F0	19 (70.4%)	13 (37.1%)	
F1	6 (22.2%)	15 (42.9%)	
F2	2 (7.4%)	7 (20.0%)	

*ESD* endoscopic submucosal dissection, *LST-G* laterally spreading tumors-granular type, *LST-NG* laterally spreading tumors-nongranular type, *LST-G-H* laterally spreading tumors-granular-homogeneous type, *LST-G-NM* laterally spreading tumors-granular-nodular mixed types, *LST-NG-FE* laterally spreading tumors-nongranular-flat elevated type, *LST-NG-PD* laterally spreading tumors-nongranular-pseudodepressed type

^a^*P* < 0.05

^b^All patients stopped the medicine for 7 days before the procedure

### Clinical outcomes

The clinical outcomes of the ESD and hybrid ESD procedures are shown in Table 2. The mean procedure time for hybrid ESD was significantly shorter than that of ESD (27.60 ± 17.21 vs. 52.63 ± 44.20 min, *P* = 0.001). However, the en bloc resection rate (77.1% vs. 81.5%, *P* = 0.677) and complete resection rate (71.4% vs. 81.5%, *P* = 0.359) of hybrid ESD were relatively lower than that of the ESD group in despite of no significant difference was found. The perforation and post-procedure bleeding rate (2.9% vs. 3.7%, *P* = 0.684) were similar between the two groups. One patient with post-procedure bleeding was successfully treated with endoscopic hemostasis therapy.

**Table 2:** Clinical outcomes

	ESD (n = 27)	Hybrid ESD (n = 35)	*P* value
Procedure time, mean ± SD, min	52.63 ± 44.20	27.60 ± 17.21	0.001^a^
En bloc resection, n (%)	22 (81.5%)	27 (77.1%)	0.677
Complete resection, n (%)	22 (81.5%)	25 (71.4%)	0.359
Clip for defect closure, n (%)	17 (63.0%)	25 (71.4%)	0.480
Successful closure, n (%)	17 (100%)	24 (96.0%)	1.000
Postprocedural bleeding and perforation, n (%)			0.684
Postprocedural bleeding	0 (0.0%)	1 (2.9%)	
Perforation	1 (3.7%)	0 (0.0%)	
Postprocedural abdominal pain and fever, n (%)	6 (22.2%)	5 (14.3%)	0.634
Additional surgery	2 (7.4%)	1 (4.8%)	0.817

*ESD* endoscopic submucosal dissection

^a^*P* < 0.05

Post-procedure abdominal pain and fever, the most common post-procedure complications, occurred in six (22.2%) patients in the ESD group and five (14.3%) in the hybrid ESD group. Clips were used to close defects in 17 (63.0%) patients in the ESD group and 25 (71.4%) in the hybrid ESD group; 17 patients (100%) and 24 (96%) patients in the ESD and hybrid ESD groups, respectively, achieved successful closure.

Two patients in the ESD group and one patient in the hybrid ESD group underwent additional surgery. No residual cancer or lymphatic metastases were discovered in the surgical specimens of these three patients. One patient in the ESD group had recurrence at a six-month follow-up colonoscopy, which was resected endoscopically. No recurrence was detected in the other patients. There was no stricture detected in the surveillance colonoscopy.

### Subgroup analysis for different endoscopic types

The results of a subgroup analysis according to endoscopic type are summarized in Table 3. There were 19 patients in the LST-G-H group, 32 patients in the LST-G-NM group, nine patients in the LSTs-NG-FE group, and two patients in the LST-NG-PD group. The LST-G-NM group had a higher rate of dysplasia and cancer than the LST-G-H group (37.5% vs. 10.5%, respectively, *P* = 0.037). The submucosal fibrosis in LSTs-NG group was higher than that of LST-G group (81.8% vs. 41.2%, *P* = 0.014). No other statistically significant differences were noted in the subgroup analysis.

**Table 3:** Lesion characteristics and outcomes of different endoscopic type

	LST-G			LST-NG				*P*
	LST-G-HG (n = 19)	LST-G-NM (n = 32)	*P*	LST-NG-FE (n = 9)		LST-NG-PD (n = 2)	*P*	
*Lesion characteristics*								
Size, mean ± SD, mm	24.26 ± 5.86	27.16 ± 8.89	0.116	24.22 ± 5.04	23.5 ± 2.12		0.803	0.544
Appendiceal orifice or ileocecal valve involvement, n (%)	4 (21.1%)	12 (28.2%)	0.221	2 (22.2%)	0 (0.0%)		1.000	0.611
Histopathology, n (%)			0.037^a^				1.000	0.822
Adenoma with low grade dysplasia	17 (89.5%)	20 (62.5%)		6 (66.7%)	1 (50.0%)			
Adenoma with high grade dysplasia + cancer	2 (10.5%)	12 (37.5%)		3 (33.3%)	1 (50.0%)			
Submucosal fibrosis, n (%)			0.831				1.000	0.014^a^
F0	13 (68.4%)	17 (53.1%)		2 (22.2%)	0 (0.0%)			
F1 + F2	6 (31.6%)	15 (46.9%)		7 (77.8%)	2 (100%)			
*Outcomes*								
Procedure type, n (%)			0.539				0.936	0.387
ESD	10 (52.6%)	14 (43.8%)		3 (33.3%)	0 (0.0%)			
Hybrid ESD	9 (47.4%)	18 (56.2%)		6 (66.7%)	2 (100%)			
Procedure time, mean ± SD, min	36.42 ± 39.82	44.84 ± 34.58	0.116	25.33 ± 9.91	16.00 ± 8.48		0.238	0.071
En bloc resection, n (%)	18 (94.7%)	23 (71.9%)	0.104	6 (66.7%)	2 (100%)		0.936	0.874
Complete resection, n (%)	17 (89.5%)	23 (71.9%)	0.260	6 (66.7%)	1 (50%)		0.618	0.515
Post-procedure abdominal pain and fever, n (%)	4 (21.1%)	6 (18.8.5%)	0.841	1 (11.1%)	0 (0.0%)		1.000	0.694

*ESD* endoscopic submucosal dissection, *LST-G* laterally spreading tumors-granular type, *LST-NG* laterally spreading tumors-nongranular type, *LST-G-H* laterally spreading tumors-granular-homogeneous type, *LST-G-NM* laterally spreading tumors-granular-nodular mixed types, *LST-NG-FE* laterally spreading tumors-nongranular-flat elevated type, *LST-NG-PD* laterally spreading tumors-nongranular-pseudodepressed type

^a^*P* < 0.05

### Univariate and multivariate analysis of a shorter procedure time

The results of univariate and multivariate analyses of a shorter procedure time (≤ 30 min) are shown in Table 4. The univariate and multivariate analyses showed that a shorter procedure time was associated with a lesion size of < 30 mm, no appendiceal orifice or ileocecal valve involvement in the lesion, and a hybrid ESD methodology.

**Table 4:** Univariate and multivariate analysis for procedure time ≤ 30 min

Factors	Univariate		Multivariate	
	OR (95% CI)	*P*	OR (95% CI)	*P*
Size, mm				
≥ 30	Reference		Reference	
< 30	5.314 (1.570–17.988)	0.007^a^	5.470 (1.078–27.766)	0.040^a^
Endoscopic type				
LST-G	Reference		Reference	
LST-NG	4.000 (0.785–20.372)	0.095	5.757 (0.664–49.915)	0.112
Procedure type				
ESD	Reference		Reference	
Hybrid ESD	4.911 (1.654–14.584)	0.004^a^	4.035 (1.109–14.679)	0.034^a^
Appendiceal orifice or Ileocecal valve involvement				
Yes	Reference		Reference	
Not	9.333 (2.558–34.048)	0.001^a^	7.025 (1.554–31.752)	0.011^a^

*ESD* endoscopic submucosal dissection, *LST-G* laterally spreading tumors-granular type, *LST-NG* laterally spreading tumors-nongranular type

^a^*P* < 0.05

## Discussion

Large cecal LSTs are challenging to resect endoscopically because it is difficult to maneuver the endoscope in the narrow intestinal space and the intestinal wall is thin. Additionally, large cecal LSTs have a higher risk of submucosal invasion, which requires an en bloc resection for precise histopathological evaluation. This study showed that hybrid ESD has a shorter procedure time compared with ESD procedure but relatively lower complete resection rate and en bloc resection rate than that of ESD. The results indicate that hybrid ESD may be an alternative for resecting LSTs > 2 cm in some difficult conditions such as submucosal fibrosis.

Hybrid ESD, which has the advantages of EMR and ESD, is a fast and feasible resection technique for colorectal superficial neoplasia [16, 19–21]. Despite research reporting a lower en bloc resection rate for large lesions [12, 13, 15], the hybrid ESD is preferable in some difficult situations, such as when lesions are hard to access, visualization is poor, or in cases of submucosal fibrosis. Previous studied have reported that tumor size, a cecal location, LST-NG, preoperative biopsy, invasive carcinoma were factors associated with submucosal fibrosis [24, 27–29]. Fibrosis, larger tumor size and paradoxical movement during the procedure were reported as independent factors contributing to the difficulty of colorectal ESD [30]. The results of these previous studies indicated that larger cecal LSTs may have a higher submucosal fibrosis and increased difficulty for ESD to perform. In this study, hybrid ESD was more used in lesions with submucosal fibrosis. The proportion of ESD was increasing while the proportion of hybrid ESD was decreasing as the study moving on. Hybrid ESD was more used in the early period of the study for its fesiablity. But in the late period of the study, hybrid ESD was more used in some difficult conditions such as submucosal fibrosis. This changing distribution of ESD and hybrid ESD cases may be a factor affected the outcomes of the study that the no statistical differences were found in en bloc resection and complete resection rate. The study results, including the en bloc resection and complete resection rates, are comparable to previous studies, but further prospective research is required to exclude the possible factors that may affected the outcomes.

In this study, 29% of the lesions had high-grade dysplasia, intramucosal cancer, and submucosal invasion cancer, which suggests that en bloc resection is required for cecal LSTs. Bae et al. [20] examined optimized hybrid ESD for colorectal tumors, which achieved similarly high rates of en bloc resection, complete resection, and adverse events, but had a significantly shorter procedure time as compared with ESD. This technique should be considered for the resection of large cecal LSTs. Factors associated with an en bloc or complete resection of large colorectal neoplasia during hybrid ESD that should be considered include visualization during snaring, fibrosis, and gross lesion type [31].

Lesions in the right colon, especially the cecum, are reportedly an independent and substantial risk factor for delayed post-polypectomy hemorrhage [32]. Low levels of post-procedural bleeding were reported in this study, which may be due to the use of clips to close defects [33]. A clip was used in 67.7% of the patients to achieve a successful closure rate. No obvious stricture was observed in the follow-up colonoscopy, which indicates the advantages of using a clip in resecting cecal LSTs.

Post-procedure fever and abdominal pain were notable complications in this study. Radiography was used to exclude possible post-procedural perforation. All patients were given antibiotics and nonsteroidal anti-inflammatory drugs, which suggests that it is important to clinically observe patients in the hospital post-procedure.

This study is limited by its small population size and retrospective, single-center design. The cecal LSTs has a relatively small proportion in colonic LSTs which limits the size of this study that may affect the outcomes of procedures and limits the value of the multivariate analysis. The choice of procedure type and outcomes of the cases in the early period may affected by the limited endoscopic instruments, equipment and the endosopists’ experience in different period of the study. En bloc resection and complete resection is necessary in LST-NG, ESD should be performed as the first choice than hybrid ESD. This study compared hybrid ESD and ESD, not EMR, in the treatment of large cecal LSTs. Future prospective, randomized, multicenter trials are needed to validate its safety and efficacy.

## Conclusion

In conclusion, this study indicates that hybrid ESD may be an alternative resection strategy for large LSTs with submucosal fibrosis in the cecum.

## Data Availability

The datasets used and/or analyzed during the current study available from the corresponding author on reasonable request.

## Abbreviations

EMR: Endoscopic mucosal resection
ESD: Endoscopic submucosal dissection
LSTs: Laterally spreading tumors
LST-G: Laterally spreading tumors-granular type
LST-NG: Laterally spreading tumors-nongranular type
LST-G-H: Laterally spreading tumors-granular-homogeneous type
LST-G-NM: Laterally spreading tumors-granular-nodular mixed types
LST-NG-FE: Laterally spreading tumors-nongranular-flat elevated type
LST-NG-PD: Laterally spreading tumors-nongranular-pseudodepressed type
